# Examining the interaction of different factors on pointing precision when using handheld laser pointers

**DOI:** 10.1186/s13104-022-05962-z

**Published:** 2022-03-07

**Authors:** Choi Yeung Andy Tse, Pui Wah Kong, Jun Jie Poh, Daniel T. P. Fong

**Affiliations:** 1 Department of Health and Physical Education, Education University of Hong Kong, Hong Kong, China; 2 Physical Education and Sports Science Academic Group, National Institute of Education, Nanyang Technological University, Singapore, 637616 Singapore; 3 National Centre for Sport and Exercise Medicine, School of Sport, Exercise and Health Sciences, Loughborough University, Loughborough, LE11 3TU Leicestershire UK

**Keywords:** Laser pointer, Aiming accuracy, Teaching

## Abstract

**Objective:**

Laser pointers are common teaching tools used during lessons. The pointing precision may influence the teaching effectiveness. In this study, we examined the effect of four external factors, namely aiming distance, target size, light condition and colour of the laser beam on the pointing precision.

**Results:**

Thirty participants (15 males and 15 females; age = 23.2 ± 4.3) were asked to aim at the target black circles with different sizes (diameters = 4 mm, 8 mm, 12 mm and 16 mm) from five various distances (2 m, 4 m, 6 m, 8 m and 10 m) at two brightness conditions (i.e., bright and dark) using two different coloured laser pointers (red and green). Three aiming parameters, namely number of hits, duration per hit and pointing precision were measured. Results showed that the aiming parameters were the highest with the aiming distance of 2 m and the use of green laser pointer towards larger target sizes regardless of the environmental brightness. Among all factors, aiming distance was the most important external factor that could influence pointing precision.

**Supplementary Information:**

The online version contains supplementary material available at 10.1186/s13104-022-05962-z.

## Introduction

Given its bright light emission ability and the nature of human eyes that tends to focus on a bright point of light, handheld laser pointer (sometimes termed laser pan) is widely utilized used as a signalling tool by educators for lectures and presentations [1], or as a teaching tool in surgery training [2, 3]. These applications have significantly improved the effectiveness of teaching and training. For example, teachers can walk across classroom and interact with their students while being able to point to their presentation screens or displays [4], while surgeons can indicate body landmarks or relevant areas of interest in surgery training [5]. However, a common problem of using the laser pointer is the pointing precision [6, 7]. Myers and colleagues [7] stated that the pointing precision was not easily maintained due to inherent human limitations (e.g., unsteady hands, poor spatial judgement), which may result in distractions and therefore negatively impact teaching and learning [6, 7].

Apart from the inherent human limitations, other external factors such as aiming distance, target size, light condition and colour of the laser beam may also affect the pointing precision. Nevertheless, no previous studies have examined the interactions of pointing precision with these external factors. How far should a presenter stand from the screen in order to aim accurately? Should the teacher turn on the lights of the classroom in order to aim accurately? Without a comprehensive study examining possible factors that can affect pointing precision, these questions remained unanswered. In the present study, we aimed to explore the interactions between the pointing precision and four external factors, namely aiming distance, target size, light condition and colour of the laser beam.

## Main text

### Methods

A total of thirty participants were recruited from the third author’s institution using the following inclusion criteria: (1) male or female; (2) 18–45 years of age; and (3) able to independently operate a laser pointer while standing. The exclusion criteria were: (1) aged above 45 years, or (2) unable to independently operate a laser pointer while standing. Written informed consent was obtained from participants. The study was approved by the Nanyang Technological University Institutional Review Board (IRB 2020-09-029). Demographic data of the participants is shown in Additional file 1: Table S1.

#### Data collection, measures and analysis

Green (model: Dobex DesignZ)—and red (model: Logitech R400) laser pointers were used in the present study. A digital camera (1920 × 1080 pixels, 24 fps; Canon G15 Power Shot) was used to record the pointing performance of the participants throughout the study. And a luxmeter (Walfront Smart Sensor AS803) was set up to measure the brightness of the experiment room. Four target black circles (printed on A4 size white paper) with diameters (4 mm, 8 mm, 12 mm and 16 mm) were put on a whiteboard. Five distances from the targets (2 m, 4 m, 6 m, 8 m and 10 m) were marked on the floor using measuring tapes. This distance range (i.e., from 2 to 10 m) is selected because educators commonly stand within such range from the screen according to our observation. There were two conditions of the experiment: bright and dark. For the bright condition, all the lights in the room were turned on with mean lux of 552 (SD = 103). For the dark condition, all the lights in the room were off with mean lux of 50 (SD = 18). The setup is presented in Additional file 2.

Each participant was asked to use the laser pointers of different colours to hit the target black circles as closely as possible from various distances at two brightness conditions (i.e., bright and dark). The duration of each trial was 10 s. In a standing position, participants were instructed to hit the target continuously for 10 s in each condition to their best effort. Sufficient time was provided for the participants to rest between trials. The orders of target sizes, aiming distances, laser beam colours and brightness conditions were randomized among all participants to minimize learning and fatigue effects. Each participant completed 80 trials in total (4 target sizes × 5 distances × 2 laser beam colours × 2 light conditions).

From the video recordings of the laser beam trajectories, three parameters of aiming performance were measured in each 10-s trial: (1) number of hits; (2) duration per hit and 3) pointing precision (i.e., percentage of the total duration of hits over 10 s). For good consistency in the timing measurements, all recorded video clips were evaluated by the same researcher (J. J. Poh) using the video analysis software Kinovea (version: 0.8.15; https://www.kinovea.org/).

#### Statistical analysis

Descriptive results were first presented for all conditions due to the exploratory nature of the study. Next, a series of repeated measures Analysis of Variance (ANOVA) were conducted to examine the interaction of different factors (distance × beam colour, distance × light, distance × target size) on pointing precision. JASP (version 0.14.1, JASP Team, 2020) was used for the statistical analysis, with significance level set at p < 0.05. *Post-hoc* analysis with Holm–Bonferroni adjustments were applied where appropriate.

### Results

**Fig. 1:**
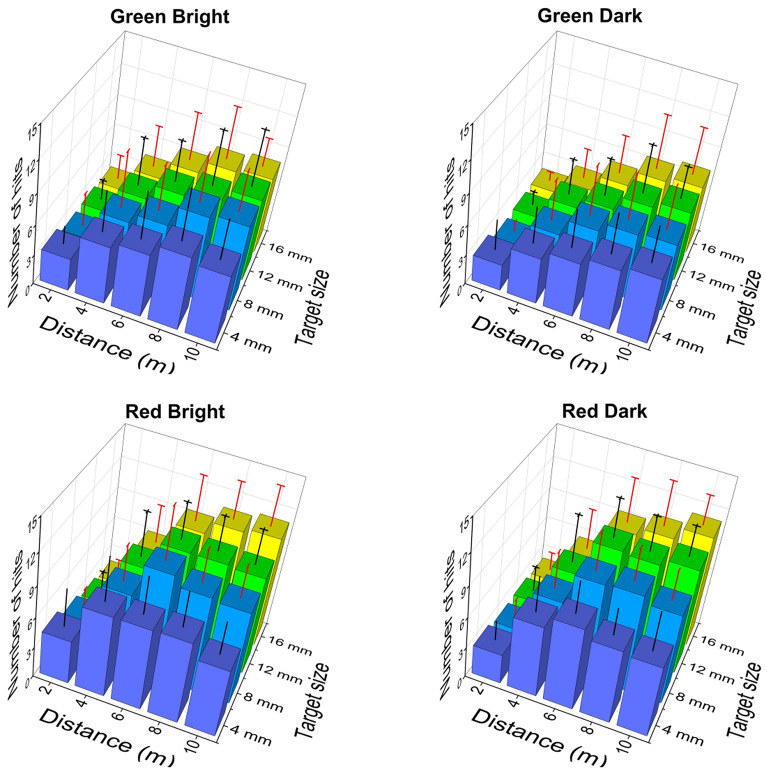
The number of hits corresponding to different conditions

Number of hits: The number of hits corresponding to different conditions (i.e., aiming distance, target size, brightness condition and colour of laser pointer) is shown in Fig. 1. As shown in the figure, it is noted that the number of hits increased with aiming distance and target size regardless of brightness condition and colour of laser beam. Comparing with different target sizes, the number of hits was the highest with the largest target size (i.e., 16 mm in diameter).

**Fig. 2:**
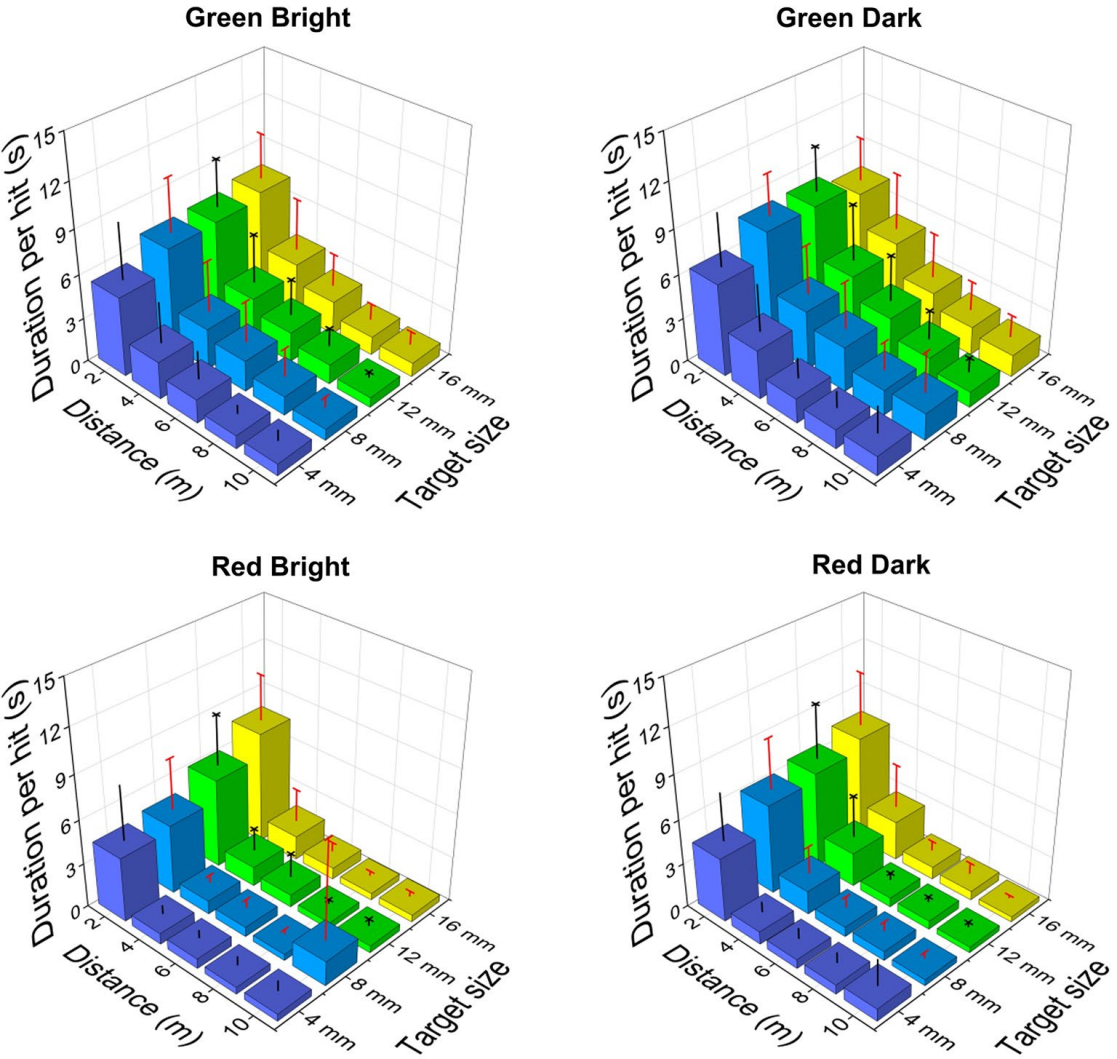
The duration per hit corresponding to different conditions

Duration per hit: The duration per hit corresponding to different conditions is shown in Fig. 2. The duration per hit decreased with increasing aiming distance regardless of the target size, brightness condition and colour of laser beam. Drastic decrease in the durations were observed when the distance increased from 2 to 4 m in all conditions.

**Fig. 3:**
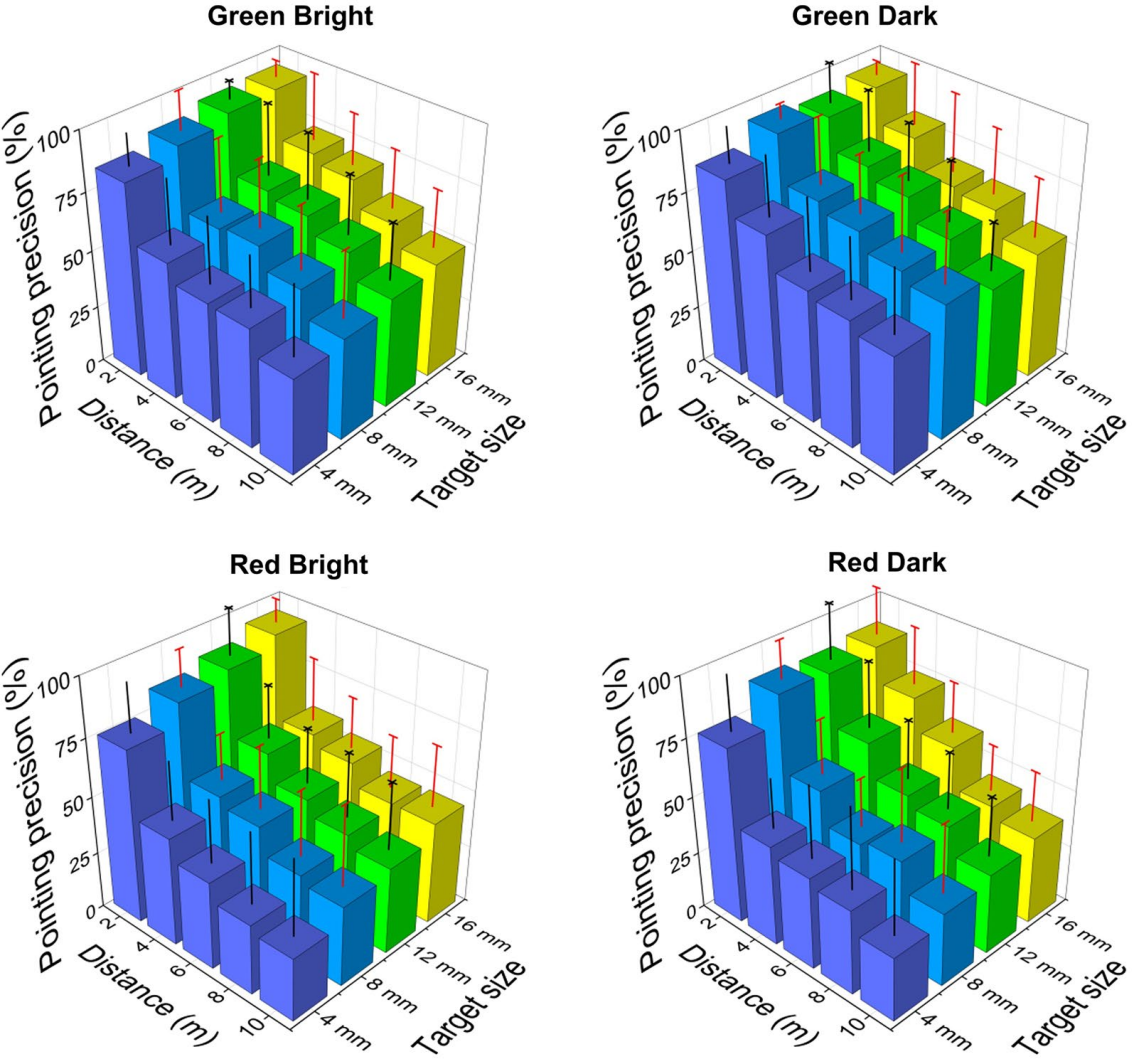
The pointing precision corresponding to different conditions

Pointing precision: The pointing precision corresponding to different conditions is shown in Fig. 3. Statistical results showed for a fixed target size (16 mm) and beam colour (green), the pointing precision significantly decreased with increasing aiming distance (*p* < 0.001, *η*^*2*^*p* = 0.548). There was no difference between the bright and dark conditions (*p* = 0.174, *η*^*2*^*p* = 0.063). *Post-hoc* analysis revealed that pointing from 2 m was more accurate than all other distances (*p* < 0.001), while other pairwise differences were less obvious (4 m > 8 m, 10 m; 6 m > 10 m; 8 m > 10 m). Regarding the effect of laser beam colour, the pointing precision of green beam was significantly higher than that of the red beam (*p* < 0.001, *η*^*2*^*p* = 0.617) across all distances for a given target size (16 mm) and brightness (bright). Finally, there was a significant effect of target size (*p* < 0.001, *η*^*2*^*p* = 0.546) across all distances with no target × distance interaction. *Post-hoc* analysis showed that pointing at the smallest 4 mm target was significantly less accurate than 12 mm and 16 mm (*p* < 0.01) for a given brightness (bright) and beam colour (green). The 8 mm target was also less accurate than the 16 mm target (*p* = 0.014).

### Discussion

The purpose of the study was to explore whether pointing precision of laser pointer were influenced by with four external factors, namely aiming distance, target size, brightness condition and colours of laser beam. Overall, the results showed that the aiming distance, laser beam colour and the target size were the major factors for the pointing precision. Importantly, the aiming distance of 2 m appeared to be critical for the pointing accuracy as the present study showed that all parameters have dropped substantially when the aiming distance increased beyond 2 m. This finding is in line with Myers et al.’s study [7], which concluded that interaction between laser pointer and computer display tended to be imprecise and error-prone with the increasing aiming distance and decreasing screen size [7].

Regarding to the colour of the laser beam, all aiming parameters were generally higher than in green laser beam condition that those in red laser beam condition across all aiming distances, target sizes and brightness settings. One possible explanation may lie on the difference in colour perception by human eyes. Studies showed that human eyes perceive green colour better than any colour of the spectrum [8, 9]. Galang and colleagues [8] suggested that participants tended to have a better estimation of laser trajectory and laser endpoint when green coloured laser beam was used. Regarding the brightness of the room, the present study showed no statistical difference in pointing precision and that all aiming parameters were similar under the rather extreme bright and dark conditions. This finding suggests that under normal classroom condition, brightness may not be a dominant factor limiting the aiming performance of the teacher. Our study, however, did not evaluate if students respond and focus better with the red or green laser beam. Future research is warranted to further investigate in this aspect.

## Limitations

Several limitations exist in the present study that require attention in future investigations. First, there is a lack of measurement of laser beam trajectory. Without such measurement, it is difficult to explain why there would be a difference of the pointing precision between different lighting, target size and distance conditions. Second, this study is constrained by one single trial for each aiming performance considering the large volume of data (80 trials per participant). While learning effect of the aiming performance should be minimized, reliability of the result is also important. Future similar studies may consider to focus on key factors and conducting additional trials for each aiming performance, and to check the test–retest reliability on different days. Third, we did not measure the angle of wrist of participants when they were aiming the target, which may confound the relationship between distance and accuracy. Future study should follow the protocol by Myers et al.’s [7] study that measured the angle of wiggle (i.e., the angle of wrist of participant in this case). In this way, we could then conclude whether the diameter of target would be promotional to the distance. Finally, the current study design could not indicate whether 2 m is the optimal distance for educators to stand from the screen for the best laser-pointing accuracy. It could simply be due to the fact that 2 m is the shortest distance measured in the present study. Future work can determine an optimal window for high pointing precision when using a handheld laser pointer.

## Supplementary Information

**Additional file 1: Table S1.** Demographic statistics of participants (n = 30).

**Additional file 2.** Set-up of the experiment.

## Data Availability

The dataset generated and analysed during the current study is publicly available and can be accessed via the NIE Data Repository (https://doi.org/10.25340/R4/BDQBVF).
