# Supplementary material for: Examining the interaction of different factors on pointing precision when using handheld laser pointers
Source: BMC Res Notes. 2022 Mar 7;15:93. doi: 10.1186/s13104-022-05962-z (PMC8900378; doi:10.1186/s13104-022-05962-z)
Supplement: Supplementary file 1 — Additional file 1: Table S1. Demographic statistics of participants (n = 30). [file 13104_2022_5962_MOESM1_ESM.doc]

Additional file 1: Table S1. Demographic statistics of participants (n = 30)

| Demographic variables | Numbers |
| --- | --- |
| Sex | 15 men, 15 women |
| Age (years) | 23.2 ± 4.3 |
| Hand dominancy (n)  Left  Right | 4  26 |
| Laser pointer use frequency (n) |  |
| Very often (at least 3 times a week) | 0 |
| Quite often (about once a week) | 1 |
| Sometimes (about once a month) | 2 |
| Occasionally (less than 3 times per year) | 21 |
| Never used before | 6 |
| Optical aids (n)  Spectacles  Contact lens | 12  1 |
| Spectacles and contact lens | 7 |
| None | 9 |
| Others* | 1 |

*One participant reported LASIK (laser-assisted in situ keratomileusis) which is an outpatient surgical procedure used to correct myopia.
