# Supplementary figures and images for: Examining the interaction of different factors on pointing precision when using handheld laser pointers
Source: BMC Res Notes. 2022 Mar 7;15:93. doi: 10.1186/s13104-022-05962-z (PMC8900378; doi:10.1186/s13104-022-05962-z)

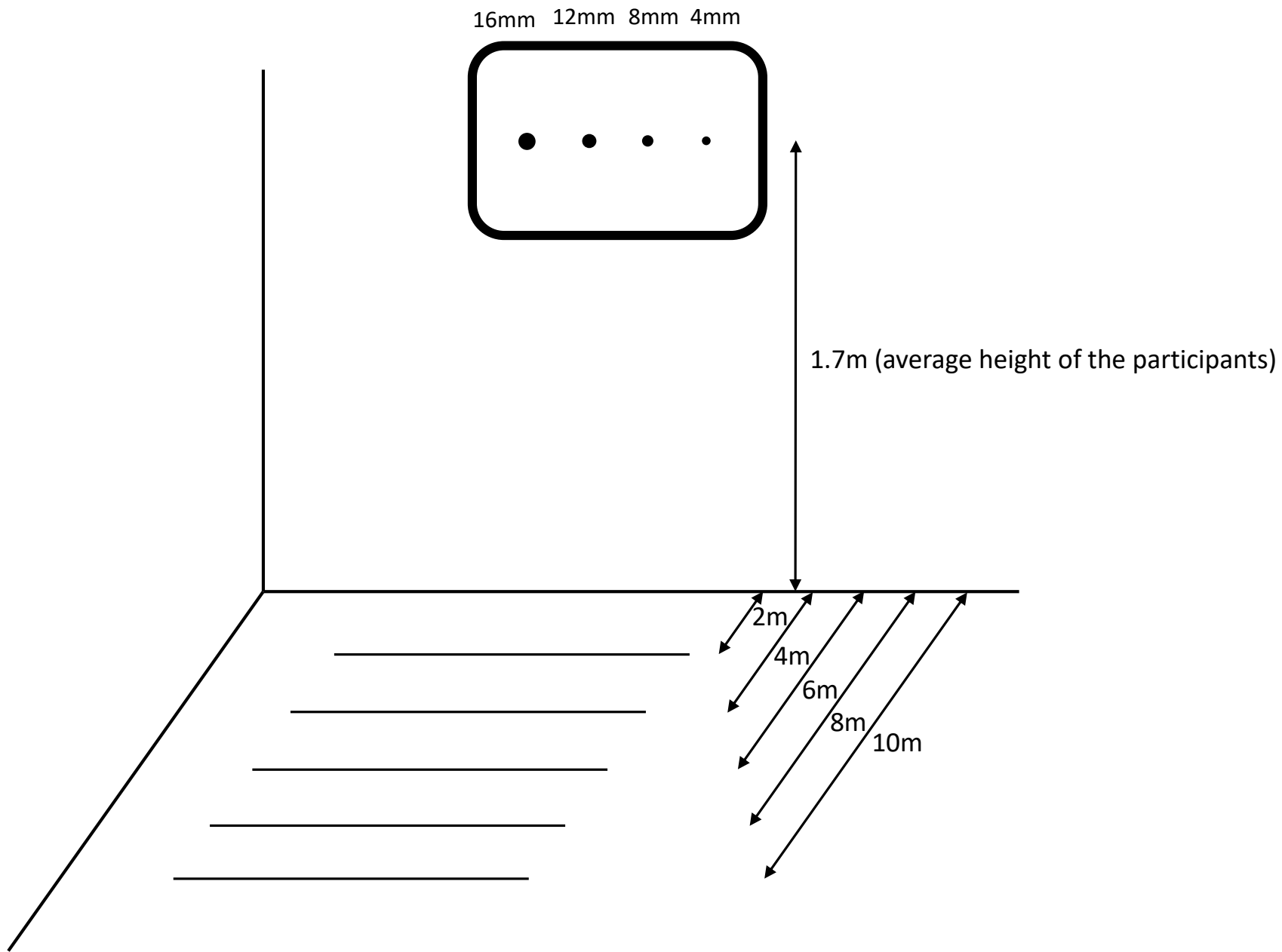

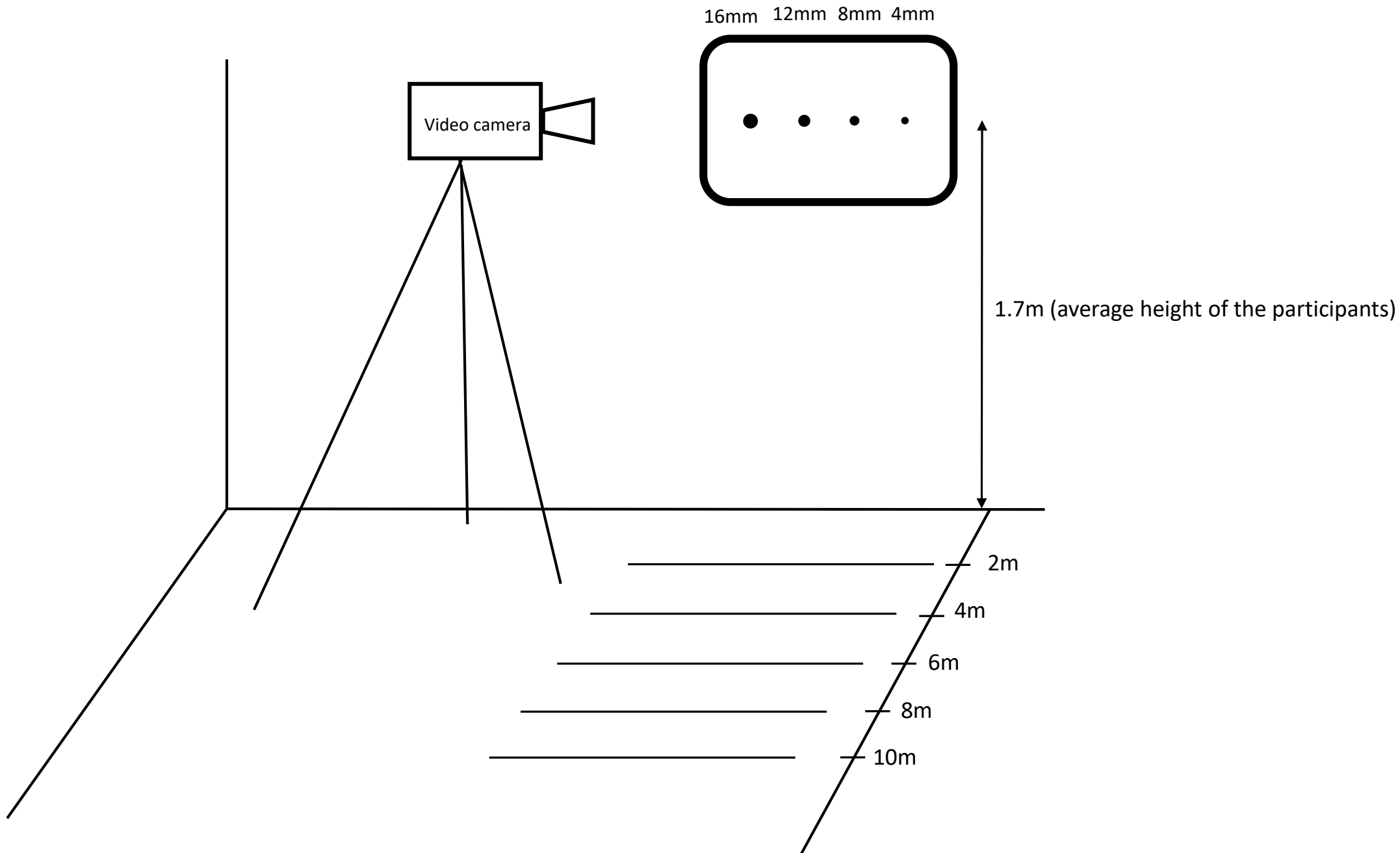

Supplement: Supplementary file 2 — Additional file 2. Set-up of the experiment. [file 13104_2022_5962_MOESM2_ESM.pdf]
